# Microfabricated polymer-metal biosensors for multifarious data collection from electrogenic cellular models

**DOI:** 10.1038/s41378-023-00488-1

**Published:** 2023-03-01

**Authors:** Charles M. Didier, Julia F. Orrico, Omar S. Cepeda Torres, Jorge Manrique Castro, Aliyah Baksh, Swaminathan Rajaraman

**Affiliations:** 1 NanoScience Technology Center, University of Central Florida, 4353 Scorpius Street, Research I, Suite 231, FL 32816 Orlando, USA; 2 Burnett School of Biomedical Sciences, University of Central Florida, 6900 Lake Nona Blvd, FL 32827 Orlando, USA; 3 Department of Biomedical Engineering, Polytechnic University of Puerto Rico, 377, 00918, Ponce de Leon, San Juan, Puerto Rico; 4 Department of Electrical and Computer Engineering, University of Central Florida, 4238 Scorpius Street, FL 32816 Orlando, USA; 5 Department of Materials Science and Engineering, University of Central Florida, 12760 Pegasus Drive, Engineering I, Suite 207, FL 32816 Orlando, USA

**Keywords:** Biosensors, Electrical and electronic engineering, Bionanoelectronics

## Abstract

Benchtop tissue cultures have become increasingly complex in recent years, as more on-a-chip biological technologies, such as microphysiological systems (MPS), are developed to incorporate cellular constructs that more accurately represent their respective biological systems. Such MPS have begun facilitating major breakthroughs in biological research and are poised to shape the field in the coming decades. These biological systems require integrated sensing modalities to procure complex, multiplexed datasets with unprecedented combinatorial biological detail. In this work, we expanded upon our polymer-metal biosensor approach by demonstrating a facile technology for compound biosensing that was characterized through custom modeling approaches. As reported herein, we developed a compound chip with 3D microelectrodes, 3D microfluidics, interdigitated electrodes (IDEs) and a microheater. The chip was subsequently tested using the electrical/electrochemical characterization of 3D microelectrodes with 1 kHz impedance and phase recordings and IDE-based high-frequency (~1 MHz frequencies) impedimetric analysis of differential localized temperature recordings, both of which were modeled through equivalent electrical circuits for process parameter extraction. Additionally, a simplified antibody-conjugation strategy was employed for a similar IDE-based analysis of the implications of a key analyte (l-glutamine) binding to the equivalent electrical circuit. Finally, acute microfluidic perfusion modeling was performed to demonstrate the ease of microfluidics integration into such a polymer-metal biosensor platform for potential complimentary localized chemical stimulation. Overall, our work demonstrates the design, development, and characterization of an accessibly designed polymer-metal compound biosensor for electrogenic cellular constructs to facilitate comprehensive MPS data collection.

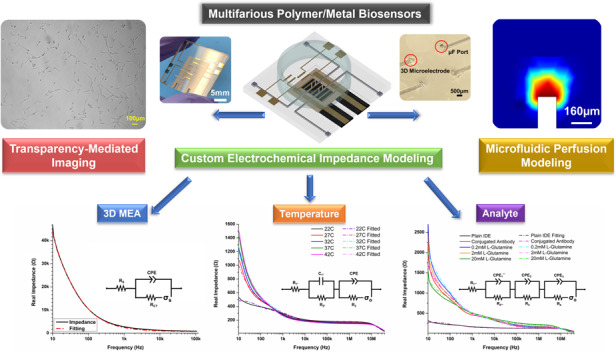

## Introduction

In vitro organ-on-a-chip models have become one of the most exciting recently emerging technologies. Located at the intersection of micro/nanoengineering and cellular biology, hybrid innovations such as on-a-chip models aim to simulate physiology at the tissue and organ level by being developing concurrently with sensing technology^1–3^. Further sophistication of such on-a-chip models requires careful crafting of organoids, spheroids, and multiaggregate cellular models to better replicate the body’s systems in vitro^4–6^. Such tissue cultures have been developed for benchtop investigation and historically have provided major breakthroughs in biological research^1^. However, these complex cellular models also require multiplexed sensing systems and strategies to procure multifarious datasets over long-term integration^7,8^.

Recently, hybrid, noncleanroom-based fabrication strategies have been employed in the fabrication of biosensors, especially with polymer-based approaches^2,5,9^. These unique approaches provide a multitude of benefits from a functional-device perspective, including easily interchangeable additive and subtractive fabrication methods and the integration of cost-effective substrate materials for optical clarity (crucial for transmitted light microscopy, a staple method in cell biology). This retention of transparency is desired in such benchtop devices to facilitate standard observational methods for comparative analysis of the discussed specialized sensing techniques. Therefore, transparent polymer substrates are possible candidates for easy integration in combinatorial additive and subtractive microfabrication steps^5,10,11^.

With respect to compound sensing modalities, electrical impedance^2^, electrochemical^12^, and electrophysiological^13,14^ measurements utilizing similar readout methodologies (such as impedimetric frequency sweeps) increase the collection of data from a single platform chip. Three-dimensional (3D) microelectrodes are a common component of next-generation toolsets for cellular interrogation^4,15^. These tools transduce voltage and current signals from electrically active cells and cellular constructs to provide readily available, functional metrics for assessing cellular health, proliferation and activity (either spontaneous or elicited). Additional electrode configurations for such measurements (e.g., interdigitated electrodes (IDEs)) may be adapted for increased sensitivity and utilization in sensing multiple signatures from cellular models^12,16^. This includes temperature and key-analyte concentration monitoring, which is highly desirable for intended cellular biosensing applications. As an example, precise control over temperature variations within the cell culture ensured one aspect of cellular homeostasis during measurements and alternatively was utilized to thermally stimulate cells when desired^17^. Moreover, the integration of an analyte-sensitive sensor into a multiplexed chip platform measured real-time, label-free reactions during an assay as electrophysiological and temperature measurements were being procured^18^. These additional sensing/application methods can be integrated seamlessly into complex “on-a-chip” biosensors, especially when much of the cell culture examination takes place outside an incubator environment (potentially leading to drastic, localized temperature variations)^19^ and when it becomes necessary to analyze changes in a cellular population longitudinally (analyte/nutrient depletion)^20^.

Concurrently, microfluidics may aid in these multifarious measurements by integrating 2D/3D ports that can perfuse compounds and/or drugs for local chemical stimulation or nutrient supplementation^3^. Such microfluidic integration may additionally engage directly with complex spheroidal organoid populations and chemically stimulate these populations locally to present new avenues for cellular growth and attachment as well as for precision placement within the culturing area itself^5^.

In this work, we demonstrate a compact, microfabricated polymer-metal biosensor platform for utilization in various “on-a-chip” applications. We demonstrate full integration of 3D microelectrodes, 3D microfluidics, IDE sensors, and a microheater, all on a single chip, and focus on impedimetric-based data analysis, as it can enable additional modalities for multifarious biosensor data analytics through frequency-dependent underlying circuit characteristics^21,22^. First, 3D microelectrode impedance characterization, as well as root mean square (RMS) noise levels, are investigated and modeled to indicate suitability for future electrophysiological measurements^23^. Next, differential temperature recordings are modeled and demonstrated using thin-film IDEs that are coupled to the resistive microheater, which is tuned to temperatures in the physiologically relevant range. A simplified plasma-enhanced conjugation regimen is additionally employed, and anti-l-glutamine antibodies are utilized in analyte detection using another integrated IDE. Sophisticated impedance modeling is utilized to identify differential equivalent circuits and extract resultant parameters for the IDE impedimetric data for both temperature and analyte detection. Standard biocompatibility and optical visualization performance of the selected materials utilizing C2C12 myocytes is presented in the supplementary data. Finally, we present COMSOL finite-element modeling (FEM) of microfluidic ports to demonstrate precise perfusion capabilities in a localized area. Together, these polymer-metal biosensor compound chip characterizations demonstrate the power of hybrid microfabrication in combination with modeling for the measurement of multiplexed datasets from electrogenic cellular models.

## Results and discussion

### Microfabrication results

**Fig. 1:**
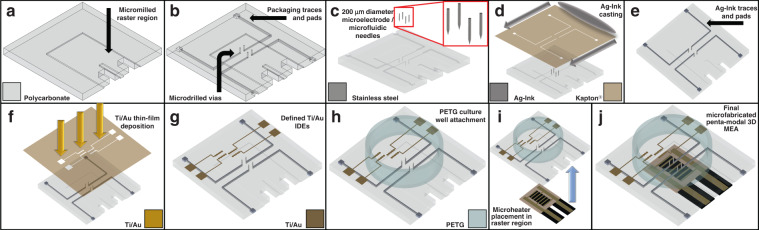
Process flow schematic for the fabrication of the polymer-metal platform. **a** Micromilled bulk polycarbonate (PC) with a raster-region with a thin cut-out for microheater placement. **b** Micromilling and microdrilling of packaging traces, pads, and through-vias, respectively. **c** Magnetic-assisted insertion of microneedles to define the bulk 3D microelectrode/microfluidic port structures. **d** Precision silver (Ag)-ink casting of the traces, pads and vias shown in **c**, utilizing a laser micromachined Kapton^®^ mask. **e** Final cured Ag-ink, defining the functional packaging for the 3D MEA sensing modality. **f** Thin-film, electron beam deposition of titanium (Ti)/gold (Au) IDEs and contact pads, for the IDE definition. **g** Final defined IDEs on the top surface of the polycarbonate substrate. **h** PET-G culture well attachment with 10:1 polydimethylsiloxane (PDMS). **i** Microheater placement into the raster-region on the underside of the substrate. **j** Final fabricated polymer-metal platform, illustrating the facile integration of multiple components in a compact fashion

**Fig. 2:**
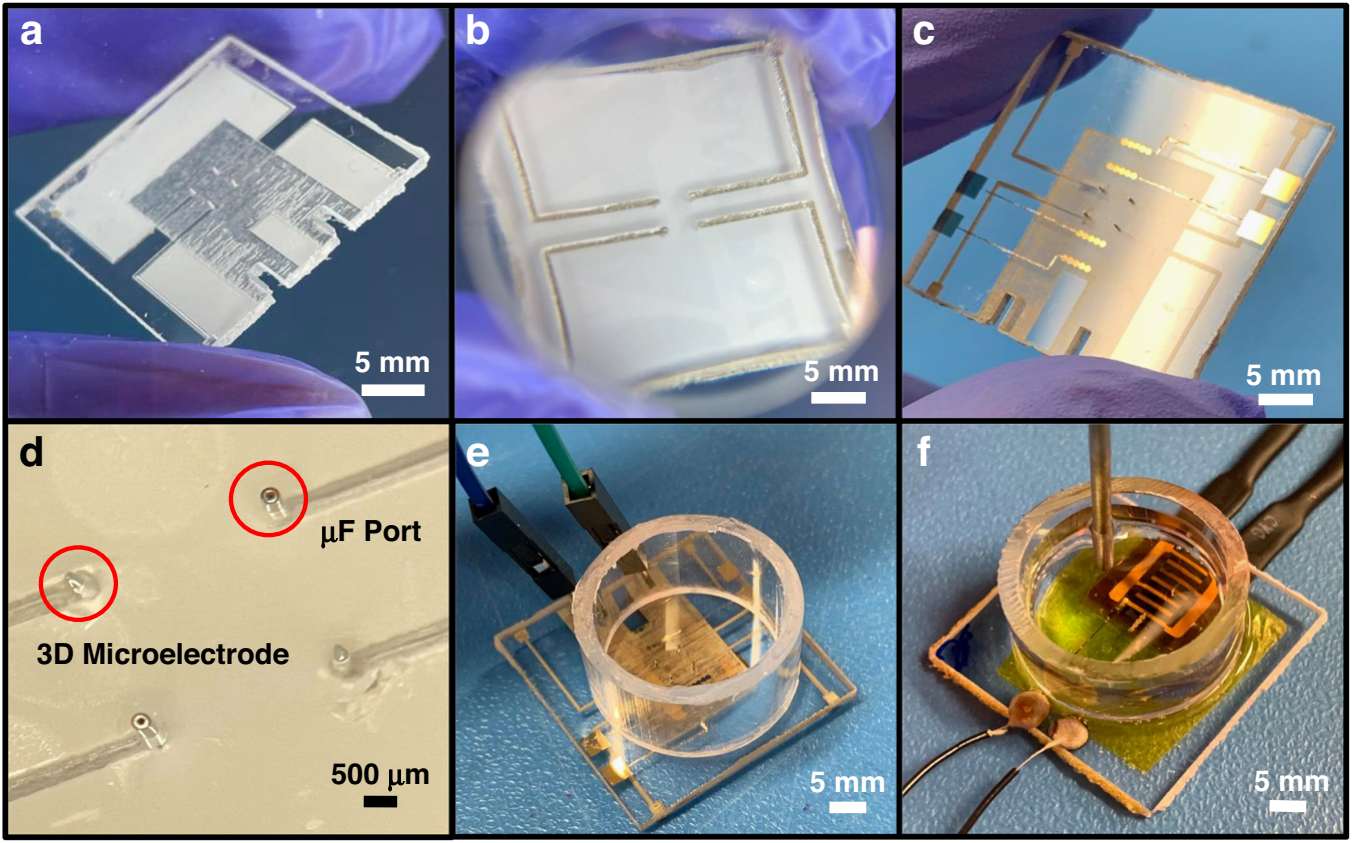
Optical images detailing selected microfabrication stages of the platform from Fig. 1. **a** Optical image corresponding to Fig. 1b. In this image, the protective film is left partially in place to better contrast each micromilled, microdrilled, or microrastered element. **b** Optical image corresponding to Fig. 1e. The cured Ag-ink is precision-aligned to reside within the desired packaging traces and pads. **c** Optical image corresponding to Fig. 1g. Both Ti/Au IDEs for the thermal and analyte sensor are deposited, and ink-cast tracing leading to the inserted 3D microelectrodes can be observed, along with the microrastered microheater region. **d** Close-up optical image of the central device region shown in **c**. Microfluidic (µF) ports are a facile substitution or addition to the 3D microelectrode insertion vias, and additional microfluidic ports may be added elsewhere in the culturing area. **e** Optical image of the culture well attachment fabrication step shown in Fig. 1h. **f** Optical image of a fabricated biosensor shown in Fig. 1j. In this image, the microheater is attached in the microrastered region for testing the devices’ temperature sensing capabilities

Figures 1 and 2 depict the microfabrication process flow for the compound chip, and optical micrographs show different stages (Fig. 2a–f) of chip microfabrication. The base microfabricated chip is shown in Fig. 2a, and the ink casting of the microelectrode packaging is shown in Fig. 2b. Figure 2c shows the final functionalization of the chip, while Fig. 2d shows an enhanced image of the central region of the chip, detailing the integration of both 3D MEA structures and metallic microfluidic ports. Additionally, Fig. 2e, f shows the integration of the microheater for temperature variation experimentation. It is desirable to enable detailed customization of transparent substrate materials to incorporate multiplexed modalities on a chip, which allows the transparency to be maintained. To this end, polymers serve as attractive materials, as they are largely biocompatible and meet optical clarity requirements while maintaining the ability to be easily modified through subtractive and additive micromachining. Additionally, the polymer utilized in this research (polycarbonate) has some noteworthy attractive properties, including resistance to cracking and well-known biocompatibility with respect to a host of cell lines^5,24^.

### Imaging and microscopy

#### SEM and AFM imaging

**Fig. 3:**
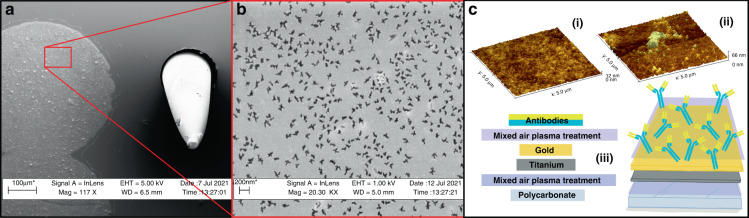
SEM images and accompanying schematic demonstrating the facile surface modification for antibody-mediated analyte detection. **a** SEM image of the Ti/Au IDE on the PC surface, along with a 3D microelectrode structure. **b** SEM image of the IDE with a higher magnification, showing the conjugated antibodies on the surface. **c** AFM imaging and schematic illustration of the image shown in **b**. (i) The functional Au IDE surface before antibodies are added. (ii) The surface of the IDE after antibody conjugation. (iii) A schematic illustration showing the overall fabrication of the IDE on PC as the base substrate, followed by a mixed-air plasma treatment, Ti seed layer, Au functional layer, and then antibody conjugation facilitated by a supplemental mixed-air plasma treatment

Figure 3a depicts an SEM image of the Ti-Au IDE configuration along with a single 3D microelectrode of the assembled device. An SEM image of the plasma-conjugated antibodies on the Au IDE surface is shown in Fig. 3b. Additionally, AFM images of the IDE are shown in Fig. 3c. The unconjugated surface of the IDE, shown in Fig. 3c (i), has a lower surface roughness (<12 nm), predominantly due to the evaporated metal. This is in contrast with the surface conjugated with antibodies shown in Fig. 3c (ii). A larger average roughness (<66 nm) is shown in this image, including a large grouping of antibodies. These results show the functionalization of the IDE surface, and the layered fabrication of the analyte sensor is schematically represented for illustrative purposes in Fig. 3c (iii). Furthermore, these results also illustrate how a simple plasma treatment regimen can be used to avoid complex conjugation chemistries. However, one disadvantage of this method is the irregularity of drop casting the antibody solution without any conjugation chemistries. While simple and effective, for more sensitive applications, a self-assembled monolayer (SAM)-mediated approach may be necessary to achieve optimized surfaces and reaction rates^25^.

#### Fluorescence microscopy

Supplemental Figure S1a–c shows the optical and fluorescent confocal microscopy images of a C2C12 culture grown on devices for 5 days in vitro (DIV). These images obtained using transmitted light microscopy illustrate the ability of these devices to retain optical clarity, even after the incorporation of all modalities of sensing (3D microelectrodes, 3D microfluidics, IDEs, and microheater). This result also shows that the polymer (PC) is suitable for transmission of both excitation wavelengths necessary to utilize calcein AM and propidium iodide. The calcein AM/propidium iodide stain ratios illustrate that the biocompatibility of the chosen material set with respect to C2C12 cells is excellent (>97%, *N* = 4).

### Electrical characterization and modeling

#### 3D microelectrode modeling

**Fig. 4:**
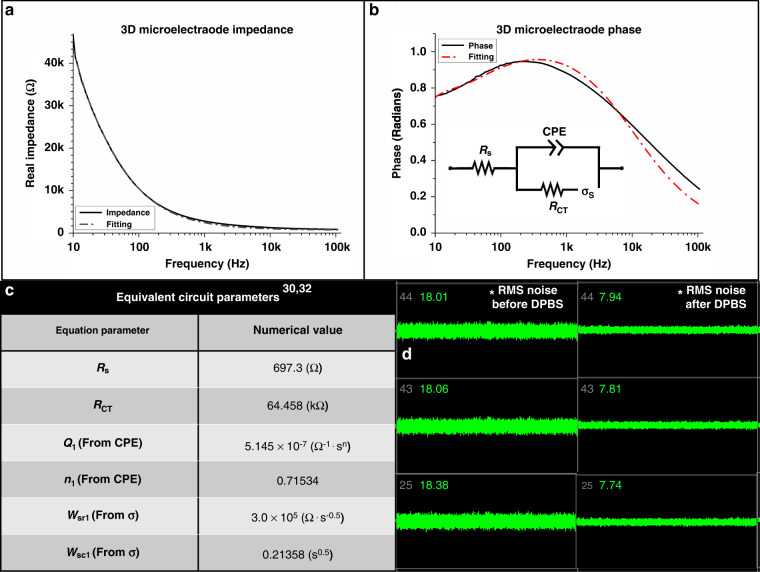
Electrical impedance characterization of the 3D microelectrode structures. (**a**, **b**) Full spectrum impedance and phase measurements. The 1 kHz impedance of 2.76 kΩ, and a phase signature of −55° are well within literature defined values. This phase signature implies a more capacitive dominance of the circuit at 1 kHz, however examination of the Nyquist plot for this data is necessary to precisely determine other properties of the 3D microelectrode. The inset of **b** contains the equivalent circuit utilized in fitting the data, and the resulting parameters are listed in **c**. **c** The equivalent circuit utilized here was a slightly modified Randles circuit, replacing the double layer capacitance (*C*_DL_) with a CPE. **d** Sample channels from the Axion MUSE® electrophysiology recording system, showing RMS noise profiles of the microelectrode/Ag-ink combination. The overall averaged RMS noise was reduced from 18.26 µV to 7.8 µV upon addition of an electrolyte solution, suitable for electrophysiology

Figure 4 shows the full spectrum impedance and phase results for the 3D microelectrodes (*N* = 4). The 1 kHz impedance of 2.76 kΩ and phase signature of −55° are well within the value ranges reported in the literature^26,27^. Utilizing a custom Randles circuit^28^, the data are fitted to extract relevant circuit parameters, such as the solution resistance (*R*_S_), charge transfer resistance (RCT), double layer capacitance (CDL), and Warburg element (*σ*) (Fig. 4a–c). Based on our recent work, the accuracy of the fitting process is enhanced by incorporating a constant phase element (CPE) in place of the standard *C*_DL_ element^29,30^.

Equation 1 is the necessary equation for defining the CPE, while Eqs. 2 and 3 list the Warburg-open (*σ*_o_) and Warburg-short elements (*σ*_s_), respectively^31,32^. Equation 4 is the algebraic representation of the equivalent circuit utilized for fitting the 3D microelectrodes, and the extracted parameters are listed in Fig. 4c.

Impedance of CPE^31–33^:

1 $$Z\left( \omega \right) = Q^{ - 1}(j\omega )^{ - n}$$

Impedance of σ_o_ and σ_s_^31,32^:

2 $$Z_{W_o}\left( \omega \right) = \frac{{W_{or}}}{{\sqrt \omega }}(1 - j)\coth [W_{oc}\sqrt {j\omega } ]$$

3 $$Z_{W_s}\left( \omega \right) = \frac{{W_{sr}}}{{\sqrt \omega }}(1 - j)\tanh [W_{sc}\sqrt {j\omega } ]$$

where:

$$\begin{array}{l}W_{or}\;or\;W_{sr} = Warburg\;Coefficient,\\ W_{oc}\;or\;W_{sc} = \frac{d}{{D^{0.5}}},\\ d = Nernst\;Diffusion\;Layer\;Thickness,\\ D = Diffusion\;Coefficient.\end{array}$$

Impedance of 3D microelectrodes:

4 $$Z_{MEA} = \frac{{R_S\left( {Z_1 + Z_{CPE}} \right) + [\left( {Z_1} \right)Z_{CPE}]}}{{Z_1 + Z_{CPE}}}$$

where:

$$Z_1 = R_{CT} + Z_{\sigma _s}.$$

The values and fitted signature imply a more capacitive dominance of the circuit at 1 kHz, which we experimentally hypothesize is due to the usage of silver epoxy with enhanced nanoscale surface porosity to define the packaging traces^34^. It is important to state as well that while various circuit elements change the impedance and phase signatures of microelectrodes, it has not been fully established if these differential values across device fabrication approaches are large enough to impact the overall performance of the microelectrodes, although we have begun to explore these questions^30^.

To assess the feasibility of utilizing uninsulated, 100 µm 3D microelectrode towers for cellular sensing, RMS noise measurements are obtained, which are important additional data that can indicate potential performance. Figure 4d shows a representative screen capture image of a set of noise measurements of the 3D microelectrodes from before (left) and after (right) the addition of DPBS. In these instances, switching from air to DPBS as the conducting medium should result in a lower noise profile. Based on the literature, reported values <10 µV are ideal for RMS noise values, so compound cellular activity may be recorded^23^. Our microelectrodes exhibit an average RMS noise value of ~7.8 µV after the addition of DPBS, showing their potential suitability for obtaining electrophysiological recordings and stimulating voltage/current.

While this work focuses on the characterization and modeling analysis of both the 3D and IDE microelectrodes described herein, our other works detail their usage in on-a-chip and MPS electrophysiologies obtained from the integration of an MPS model with a polymer-metal biosensor platform^35^.

#### IDE temperature modeling

For impedimetrically characterizing temperature changes through an integrated IDE sensor, an investigation is warranted to determine the frequency range in which the temperature sensitivity is the highest. Since full-spectrum impedance sweeps are performed, analysis of different slices of the spectra is carried out to determine the ideal region for detecting temperature changes with the highest sensitivity. For the given application, a practical understanding of the potential for thermal loss is important. Internal calibration tests are performed and utilized prior to functional device testing (not shown), which also serves to test the supplemental thermal confirmation tools for use in validating the impedimetric readings.

**Fig. 5:**
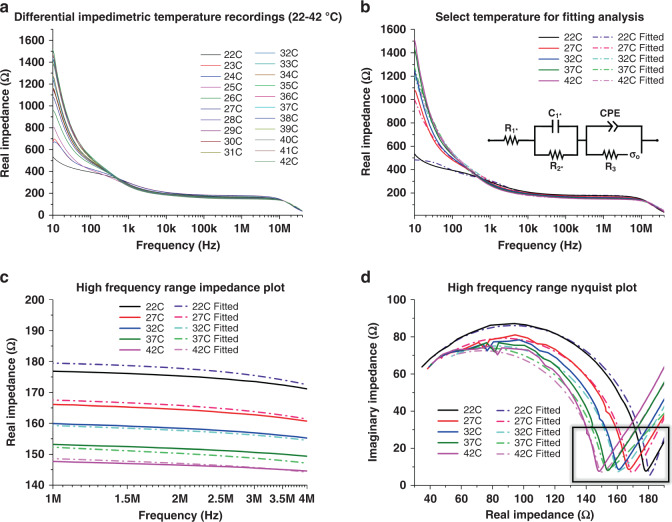
Electrochemical impedimetric characterization of the base IDE fabrication at differential temperatures, utilizing the attached microheater to vary the DPBS temperatures. **a** Compilation of all impedimetric temperature recordings across the (22–42 °C) temperature range. **b** Selected temperatures (every 5 °C) from **a**, including fitted models for each, derived from the equivalent circuit shown in the inset. **c** Enhanced inset data from **b**, showing the 1–4 MHz region of interest, where changes in the faradaic reactions at the electrode surface are apparent. Here, a clear decreasing impedance trend is observed, consistent with a hypothesis of increased kinetic activity within the electrolyte solution. **d** Nyquist plot variation of the data from **b** and **c** which plots imaginary impedance against real impedance. The curve shown corresponds to the first faradaic reactions governed by the start of the equivalent circuit, present at the higher frequencies indicated. The highlighted region of the curve is governed primarily here by R_1*_ and R_2*_ and confirms the same trend of decreasing real impedance observed in **c**

Initially, it was suggested in established literature that in a simple Randles circuit, the *R*_S_, *R*_CT_, and *C*_DL_ values govern the Faradaic, kinetic electrochemical interactions present at higher frequencies but also play a role along with the Warburg diffusion elements at lower frequencies^36,37^. In general, lower frequencies are susceptible to high levels of environmental noise, which can produce unreliable results^26^. Since the evaluation of temperature utilizing an impedimetric sensor is largely influenced by the energetic activation of dissolved ionic species in media, it is determined that higher frequencies are more likely to present a reproducible and consistent temperature change measurement^34^. As the temperatures being measured are in the physiologically relevant range (22–42 °C) and are not high enough for large evaporation to occur, a decrease in impedance is expected due to increased ionic species motility^34^. If the temperatures of interest were much higher, then an increase in impedance would be expected with resultant relative increased concentrations of ionic species from reduced solution volumes. The results shown in Fig. 5a indicate such an overall expected trend of decreasing impedances as the temperature is increased.

As discussed in the section “3D Microelectrode Modeling”, a custom equivalent circuit is utilized to fit the temperature IDE. The circuit diagram is shown in Fig. 5b, along with the impedance and Nyquist plots necessary to fully analyze the sensor configuration shown in Fig. 5c, d. The Nyquist plot is especially important, as it illustrates how a traditional Randles circuit must be modified with additional circuit elements to account for the secondary Faradaic regions observed^36,37^. It should be noted, however, that because the equivalent circuit is affected by a combination of circuit elements, especially with respect to the faradaic reactions, *R*_S_ is not the only element of consequence (as in traditional impedimetric analysis) and thus must be taken into account with both *R*_CT_ and *C*_DL_ to fully characterize the impedance changes observed at these higher frequencies.

Equation 5 is thus justifiably different than Eq. 4, as it accounts for new capacitive and resistive elements resulting from the differing circuit composition and materials. The values extracted from this parameter fitting are reported in the supplementary information (Fig. S2 and Table S1). The impedance of *σ*_o_ is calculated with Eq. 2 in section “3D Microelectrode Modeling”.

Impedance:

5 $$Z_{Temperature} = \frac{{R_{1 \ast }\left( {R_{2 \ast } + Z_c \ast } \right)\left( {Z_1 + Z_{CPE}} \right) + \left[ {\left( {R_{2 \ast }Z_{c \ast }} \right)\left( {Z_1 + Z_{CPE}} \right)} \right] + [(Z_1Z_{CPE})(R_{2 \ast } + Z_{c \ast })]}}{{(R_{2 \ast } + Z_{c \ast })(Z_1 + Z_{CPE})}}$$

where:

$$Z_1 = R_3 + Z_{\sigma _o}.$$

Based on the frequency spectra evaluation, 1 MHz-10 MHz is generally determined to be the region of interest for temperature sensing (*N* = 4). Utilizing a linear regression, the adjusted regression value (*R*^2^) of 0.97506 validates the observed consistent impedance trend decreasing across all temperatures tested. Incorporating the fitting model allows for the frequency band of interest to be narrowed to 1–4 MHz by utilizing 5 °C temperature increments, spanning the range tested (Fig. 5b, c). This frequency range is subsequently confirmed utilizing the experimental and fitted Nyquist plots, which clearly demonstrate a measurable leftward shift of the first Faradaic curve, corresponding to these higher frequency regions of interest. Specifically, this is in reference to the inverted peak highlighted in Fig. 5d, which is primarily governed by *R*_1_^*^ and *R*_2_^*^ (denoted as such here because this equivalent circuit is a modified “Randles” circuit and thus these values are not necessarily directly equivalent to *R*_S_ and *R*_CT_)^36,37^.

Finally, to confirm the inability to utilize the lower frequencies for temperature sensor measurements, linear regressions are also calculated from 10 to 100 Hz (not shown). The observed *R*^2^ value of 0.65958 supported our hypothesis that potential noise and general inconsistencies across measurements in this region produce unreliable temperature readings.

#### IDE analyte modeling

Fortunately, there are many options for developing an antibody conjugation protocol for a surface with Au as the functional layer. It has been well established that free thiol groups facilitate adsorption onto Au-metal surfaces^38,39^, and this presents opportunities for simple antibody conjugation, although potentially in a random manner^40,41^. This phenomenon is most widely reported in literature related to nanoparticles^41^; however, an evaporative metallization process, such as the electron-beam physical vapor deposition method utilized here, produces a similar high-surface-area metal layer for potential antibody binding. Plasma treatment is introduced to enhance the binding affinity of the antibodies to the Au surface, although in subverting conjugation chemistries, some minimal loss of antibodies is expected. Anti-l-glutamine antibodies are selected for characterization, as l-glutamine is a critical component in cell culture media^42^. For example, it can be applied as a potential screening target during assays because although it is a necessary component, it degrades readily into toxic ammonia, which may be utilized as an indirect indicator for necessary media changes^43^. An alternative application was demonstrated by Rubin et al., who reported that l-glutamine was important for controlling cancer cell proliferation^44^.

**Fig. 6:**
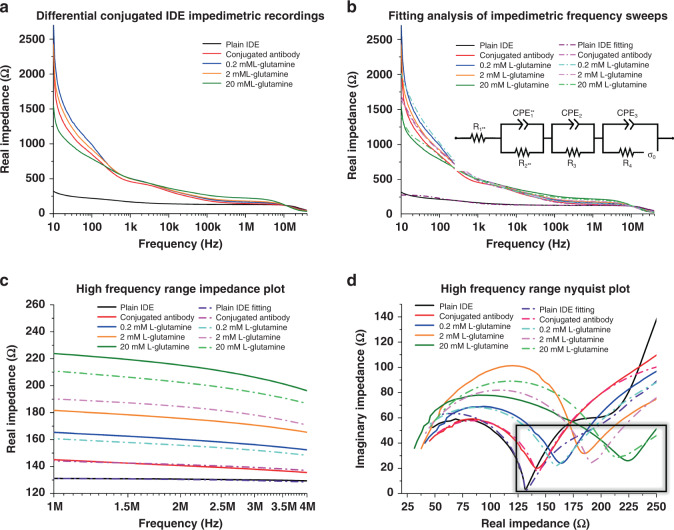
Electrochemical impedimetric characterization of the simplified fabrication of the antibody-conjugated IDE. **a** Compilation of all impedimetric recordings from the various states of the IDE. **b** Impedance data from **a**, including fitted models for each state, derived from the equivalent circuit shown in the inset. The “Plain” IDE state was fitted utilizing the equivalent circuit shown in Fig. 4b, as the fabrication of the sensor up to this point is identical. **c** Enhanced data from **b**, showing the 1–4 MHz region of interest, where changes in the faradaic reactions at the electrode surface are apparent. Here, as opposed to that shown by the temperature sensor, a clear increasing impedance trend is observed. **d** Nyquist plot variation of the data from **b** and **c** plotted as the imaginary impedance against the real impedance. The curve shown corresponds to the first faradaic reactions governed by the initiation of the equivalent circuit, present at the higher frequencies indicated. The curve here is governed by R_1**_, R_2**_, and CPE_1**_ and confirms the increase in real impedance shown in **c**. Additionally, a change in the curve shape is observed, owing to the capacitive elements imposed on the IDE surface from antibody conjugation and analyte binding

Full-spectrum impedance sweeps are performed for the analyte-sensing IDE (Fig. 6a). Similar to the temperature IDE, the expected frequency region of interest is located in the higher frequency range between 1 MHz and 4 MHz due to the functional changes expected through the conjugation of the antibodies on top of the IDE. A change in the capacitive component of the equivalent circuit is expected once the antibody is conjugated^45^, and this is confirmed both through fitting (Fig. 6b–d) and phase plots (Fig. S3).

To model the conjugating antibodies on the surface of the IDE, another CPE element in parallel with *R*_4_ is added to the equivalent circuit to incorporate additional dielectric layers in the signal pathway. This modifies the resulting equation for the analyte-sensing IDE (Eq. 6), which is simplified as shown:

Impedance:

6 $$Z_{Analyte} = \frac{{R_{1 \ast \ast }\alpha + \left( {Z_{CPE_{1 \ast \ast }R_{2 \ast \ast }}\gamma \delta } \right) + \left( {Z_{CPE_2}R_3\beta \delta } \right) + (Z_{CPE_3}Z_1\beta \gamma )}}{\alpha }$$

where:

$$\begin{array}{lll}Z_1 &=& R_4 + Z_{\sigma _o},\\ \alpha &=& \beta \gamma \delta ,\\ \beta &=& R_{2 \ast \ast } + Z_{CPE_1 \ast \ast },\\ \gamma &=& R_3 + Z_{CPE_2},\\ \delta &=& Z_1 + Z_{CPE_3}.\end{array}$$

Again, as this more complex equivalent circuit is not a traditional Randles model, the components related to the first Faradaic curve of interest are denoted with (**). This is done to highlight their relative placement in the equivalent circuit in comparison to a more traditional set: *R*_S_, *R*_CT_, and *C*_DL_, which become R1^**^, *R*_2_^**^, and CPE_1_^**^, respectively. The values for these fitted models are listed in the supplementary information (Table S2). Similarly, the impedance of σ_o_ is calculated with Eq. 2 in the section “3D Microelectrode Modeling”. The unconjugated (just Ti/Au) IDE is fitted with the same equivalent circuit as that discussed in section “IDE Temperature Modeling” because the IDE has the same fabricated structure.

As discussed in the section “IDE Temperature Modeling”, a linear regression (*R*^2^) value for these data is calculated as 0.90517, providing an affirmative indication of the effectiveness of this IDE in detecting a number of differential concentrations of l-glutamine across the 0.2 mM-20 mM range.

Examining solely the overall frequency plot of the impedance (Fig. 6a), no clear trends are observed across differing l-glutamine concentrations. However, previous impedimetric sensor studies have shown that the frequency range utilized may be tuned to the analyte of interest^46^, and the higher frequencies here may allow observation of relevant faradaic changes. Indeed, upon closer inspection of the 1 MHz-4 MHz region, a consistent pattern (*N* = 4) is observed, and this is supported both by the shift within the Nyquist plot and the fitting of the experimental readings. A well-defined increase in impedance is observed at the end of the first Faradaic curve in the Nyquist plot (which is governed here by *R*_1_^**^ and *R*_2_^**^). It is also interesting to note the change in the shape of the curve and the impact of the imaginary part of impedance as the concentration increases. This is likely due to an impact on the capacitive circuit elements (here governed by CPE_1_^**^) imposed by the addition of the antibodies and l-glutamine in solution. The probable saturation could additionally be due to the robustness of the antibody adherence to the Au surface, which successive wash steps could impact, along with the impacts of the total surface area of the IDEs (which may not be optimized for this particular analyte reaction). As suggested earlier, by utilizing SAM-based conjugation chemistry, the orientation of the antibodies can be controlled, along with the longevity of their adherence to the IDE. However, such a process introduces potentially hazardous chemicals into the integration of an MPS and increases the complexity of the defined microfabrication approach^25^. Additionally, blocking steps should be utilized in the practical application of such a conjugation, which are excluded from the experimental protocol to simplify the measurements.

### Fluidic modeling

**Fig. 7:**
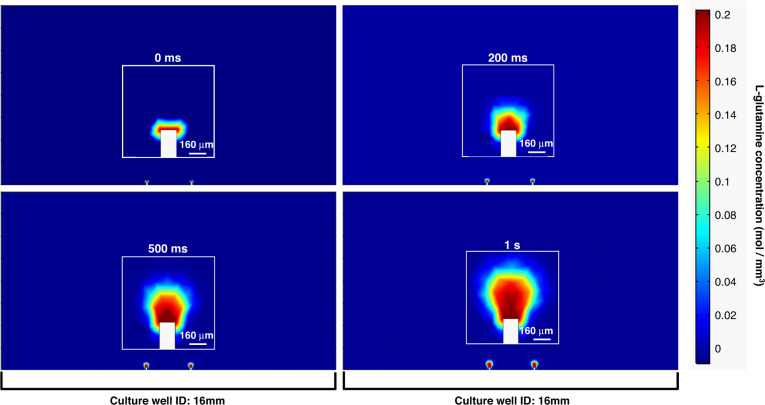
COMSOL microfluidic modeling of the device with a constant 8 Pa applied pressure. The timepoints are 0 ms, 200 ms, 500 ms, and 1 s post application. These time points are reflective of the localized application of the simulated l-glutamine. The inset images are enlargements of a port at each timepoint to better visualize the concentration changes (scale bars are for the inset images only). At 0 ms, there is no substantial ingress of the simulated 0.2 mM l-glutamine solution. At 200 ms and at 500 ms, the solution is still highly localized near the inlet. At 1 s, the solution is still localized, however this timepoint is the maximal time at which pressure should be applied to retain local chemical stimulation. Images of timepoints beyond this 1 s time are shown in Supplementary Fig. S4

Figure 7 illustrates the results of COMSOL fluidic modeling performed to assess the feasibility for the localized application of compounds in a defined culture area. As observed, with a nominal 8 Pa of force consistently applied over time, 0.2 mM L-glutamine (an example nutrient) is locally concentrated under the 1 s timepoint. Across the span of time points shown (0 ms, 200 ms, 500 ms, 1 s), the solution is controllably dispersed, which allows for precise volumetric injection into a spheroid located on top of the port^5^. With continued pressure (Fig. S4), there is an appreciable increase in diffusing volumes through the port approaching 5 s. From 10–20 s of continued application of pressure, the solution begins to disperse over a larger area within the culture, similar to dosing a culture traditionally with larger pipettes, for more global/mesofluidic chemical stimulation. The results for the continued application of pressure after 5 s are listed in the supplementary materials (Fig. S4). This highlights the necessity for low pressures, lower concentrations, and shorter (ms) chemical stimulation and demonstrates the ability to locally apply a defined compound (in this instance, l-glutamine) through simple 2D/3D microfluidic port integration. These results, however, are not indicative of any one solution for the optimal pressure, concentration, rate of flow, or time for utilizing localized microfluidic perfusion and serve only as an example of one possible combination of the parameters mentioned. Additionally, while the pressures modeled here are within the range for human actuation, syringe pumps would provide a more controlled application of force for millisecond application times.

Chemical stimulation in a localized setting, such as that modeled here, is vital, for instance, in studying the effects of localized dopamine stimulation and the effects of such stimulation on cellular populations using localized field potential measurements additionally enabled by the device^47^. Furthermore, such chemical stimulations can be tuned through optimization studies for individual uptake and diffusion characteristics of desired compounds^48^. The forthcoming application of microfluidic integration for localized perfusion and controlled spheroidal manipulation is ongoing as a continuation of our existing work^5^.

## Materials and methods

### Chip design and microfabrication

The base microfabrication process for the combination platform chip is shown schematically in Fig. 1. Using Solidworks 3D design software (Dassault Systèmes, France), a 24 mm × 24 mm polycarbonate (PC) chip was designed to be micromilled from a 1.75 mm bulk substrate. Drawing Interchange Format (DXF) files from the 3D design were imported into the T-Tech QC-J5 Quick Circuit Prototyping Systems (T-Tech, USA) for microdrilling (μD) and micromilling^22^. All parameters were standardized with a spindle speed of 60,000 rpm and a feed rate of 25 mm/s, and all depths of cut were specified individually as described. A raster pattern of 300 µm x 300 µm (L x W) with a depth of 300 µm was micromilled on the back of the chip where the microheater was to be placed, as shown in Fig. 1a. Subsequently, 1 mm×1 mm contact pads were micromilled with 200 µm wide traces to define the packaging components on the backside of the chip (Fig. 1b). Additionally, 220 μm wide and 500 µm deep vias were microdrilled (μD) in the center of the chip to place 3D microneedles for 3D microelectrode incorporated (Fig. 1b).

### 3D microelectrode definition

3D microelectrode structures were defined using ~200 µm diameter needles (DBC^TM^, South Korea). Using a 1064 nm IR wavelength, the needles were laser micromachined (QuikLaze, USA) and subsequently clipped to a final height of ~1.85 mm. The 3D microelectrodes were manually placed using a magnetic insertion process (MagIN)^5^, as shown in Fig. 1c, resulting in a functional height of the final microelectrode of approximately 100 µm. The 3D microelectrodes were packaged using conductive silver (Ag)-ink (EP3HTSMED; MasterBond, USA) casting, which was defined on top of the micromilled tracing and pads on the backside of the chip. UV-laser micromachined Kapton^®^ (DuPont, USA) shadow masks were utilized to aid in the process and ensure high specificity in deinition. The Ag ink was cured at 60 °C for 24 hours to achieve a mechanically robust profile (Fig. 1d, e).

### Interdigitated electrode definition

Shadow masks were designed using Solidworks 3D CAD software and were UV-laser micromachined from 25 μm thick Kapton^®^ sheets with a biocompatible adhesive backing layer (Fig. 1f). Titanium (Ti)–gold (Au) interdigitated electrodes (IDEs) were defined with overall dimensions consisting of a two-finger, 750 µm pitch circle-in-line geometry^16,49^. Each finger consisted of five (5) 750 µm diameter, contiguous circles, and resulted in a total functional length of 3.75 mm. Masks were subsequently aligned utilizing existing features on the topside of the chips.

IDE masks were placed adjacent to but not directly on top of the location of the microheater underneath the chip. All IDEs were fabricated utilizing electron-beam (E-beam) evaporation (100 nm Ti/200 nm Au; Thermionics, USA), as shown in Fig. 1g.

Conjugated IDEs were defined with the same Ti/Au e-beam conditions as described above utilizing known Au layers to serve as the functional interface for antibody adherence and thiol-mediated adsorption^38,39^. Anti-l-glutamine-specific antibodies (Abcam, UK) were obtained (~10 mg/ml) and were subsequently diluted down to ~0.1 mg/ml (100:1) aliquots in Dulbecco’s phosphate buffered saline (DPBS; Millipore Sigma, USA). Devices were washed with 70% ethanol (Sigma Aldrich, USA), dried and then plasma-treated (Plasma Etch, USA) for 20 s under mixed-air conditions. A measured volume of 40 µl of the diluted antibody aliquot was pipetted onto the plasma-treated Au-IDE surfaces and incubated at 22 °C for 1 h before being gently washed with deionized (DI) water and dried.

### Culture well attachment

A culture well for the device was defined using a transparent polyethylene terephthalate glycol (PETG) ring with a 16 mm inner diameter and 8 mm height, as shown in Fig. 1h. Standard 10:1 polydimethylsiloxane (PDMS) viscoelastic polymer was utilized to attach the culture well and was allowed to cure at 60 °C for 2 h to obtain the desired final properties.

### Microheater attachment

A resistive microheater (Pelonis Technologies, USA) was attached to the backside of the chip in the micromilled raster region, as shown in Fig. 1i, j. To utilize the microheater, the cathode and anode connections were wire-bonded to contact points. The microheater was powered with a source meter (Keithley, USA), holding the current compliance at 1.05 µA and varying the voltage from 0.5 V to 7 V (with 0.5 V increments). To measure the resulting temperatures to calibrate the microheater, a combination of an IR thermal camera (Perfect Prime, USA) and an external probe were used.

### 3D microelectrode characterization

Full-spectrum impedance and phase characterization were obtained with electrochemical impedance spectroscopy (EIS) on a Bode 100 impedance measurement system (Omicron Labs, Austria). Measurements were performed across the frequency range of 10 Hz to 40 MHz in DPBS with a platinum (Pt) counter electrode. Root mean square (RMS) noise measurements were obtained using the MUSE^®^ electrophysiology system (Axion BioSystems, USA).

### IDE characterization

IDE characteristics were assessed either utilizing a single IDE finger with a Pt counter electrode in DPBS (as was utilized in the 3D microelectrode characterization protocol) or between both fingers of the respective IDE. Scans were obtained across a full frequency spectrum of 10 Hz to 40 MHz.

To analyze the temperature recordings, both fingers were utilized to measure changes in DPBS. Baseline impedance spectra were recorded at 22 °C (room temperature). Impedance scans were subsequently recorded as the voltage was increased from 0.5 V to 7 V across the microheater to produce a range of physiologically relevant temperatures ranging from 22 °C to 42 °C. Changes in impedance were recorded as the temperature increased. Temperatures were validated utilizing an IR thermal camera (Perfect Prime, USA) and an additional external probe.

For surface conjugated impedance analysis, measurements were obtained in the same manner as above, with the addition of a Pt counter electrode. To obtain baseline recordings, unconjugated sensors were characterized before conjugating the antibodies as described previously. Impedance measurements were then performed again on the conjugated IDEs to assess the sensitivity of each step in the process. As an indirect measure of the surface modification of the IDEs, aliquots of l-glutamine were prepared at varying concentrations in DPBS (20 mM, 2 mM, and 0.2 mM). Between each scan and when changing the concentration, the sensors were washed with fresh DPBS for 30 s to allow the IDEs to return to baseline.

### Impedance and equivalent circuit modeling

Fitting analysis of the 3D microelectrode, temperature IDEs, and analyte IDEs was performed using EIS Spectrum Analyzer Software (EIS Spectrum Analyzer Software, Belarus), with 300 iterations for convergence of the Powell algorithm and using the amplitude function^31,32^.

### Sample imaging

Samples were imaged using a combination of atomic force microscopy (AFM) and scanning electron microscopy (SEM). AFM (Veeco dimension 3100, USA) was utilized in tapping mode with PR-EX-T125-10 tips (Resonant Frequency, 200–400 kHz; Spring Constant, 13–77 N/m; Anasys Instruments, USA). SEM (Zeiss nvision 40, Germany) imaging was performed at an incident voltage of 1–5 keV. Optical images were obtained using an iPhone XS (Apple, USA). Fluorescence and optical microscopy were performed using a Keyence BZ-X810 laser confocal microscope (Keyence, Japan). The laser confocal excitation wavelengths of the system utilized were 470 nm ± 40 (calcein AM) and 560 nm ± 40 (propidium iodide).

### COMSOL fluidic modeling

COMSOL modeling of localized microfluidic flow ingress into the culture area defined in this work (total volume of 1,608.5 mm^3^) was performed using a time dependent, laminar flow arrangement^50^ (COMSOL Multiphysics 5.4; COMSOL Inc., Sweden), and the model was calculated utilizing the finer mesh setting. The model itself was calculated as a 2D representation of the 3D fluid space to better visualize the ingress of microfluidic volumes. The fluidic ports utilized in this model were dimensioned similar to 30 G dispensing needles (BSTEAN^TM^, USA) in two-dimensional equivalents (OD: 220 µm; ID: 160 µm). The pressure applied was a nominal 8 Pa of force (to simulate a gentle pressing on a syringe), which provided more control based on maximal human-applied pressures on syringes. The liquid injected was simulated using the 0.2 mM l-glutamine solution described in this work.

### Cell culture

C2C12 murine myoblast cells (CRL-1772^™^; ATCC, USA) were cultured utilizing standard protocols on devices for 5 days in vitro (DIV). Brightfield and fluorescence microscopy were performed as described in Section 3.9, with calcein AM and propidium iodide live/dead stains (Thermo Fisher Scientific, USA) utilized for transparency and transmittance confirmation, as well as for cellular viability calculations.

## Conclusions and perspectives

In vitro organ-on-a-chip models are becoming increasingly necessary for greatly advancing studies of tissue culture models on the benchtop. By developing multimodal sensors for the integration of these models all on a chip, multifarious datasets can be procured, and differential measurements can be compared. In this study, we expanded on our polymer-metal biosensor platform^5^ through facile functionalization with thin-film IDEs to demonstrate how impedimetric analysis can allow access to new functional readouts by modulating the frequency of interest. Additionally, we fit impedance data from the 3D microelectrode structures, as well as the IDEs for two different sensing modalities with custom equivalent circuits, to illustrate how functional changes to microelectrode and interdigitated electrode fabrication impact the underlying performance of circuit parameters and begin to apply this understanding to the differential recordings presented.

The 3D microelectrodes themselves demonstrated a 1 kHz impedance of 2.76 kΩ and a phase signature of −55°, along with an RMS noise signature of ~7.8 µV, indicating that the literature supported the suitability of these microelectrodes for electrophysiological stimulation and recording applications (not shown in this work but in an upcoming article). The first IDE configuration was coupled with an integrated microheater to vary temperatures in the physiologically relevant range (from 22 °C to 42 °C) and subsequently record differential impedance readings for analysis. Through our modeling and resultant Nyquist plots, it was reported that there is an inverse relationship between the temperatures detected herein and the corresponding impedances. The indicated detection region of highest interest for localized temperature in the culture well was determined to be between 1 MHz and 4 MHz, where the elements of the equivalent circuit impacted the first Faradaic region. A linear regression of the measured temperature data produced a high correlation (R^2^ value of 0.97506), which in combination with the fitting analysis demonstrated the utility of the Nyquist analysis to not only demonstrate differential trends but also to result in supplemental parameter changes.

Next, conjugation of a desired antibody in a simplified thiol-mediated fashion was performed utilizing an anti-l-glutamine antibody. Then, a custom fitting analysis was utilized along with Nyquist plots to determine how such a simplified approach might be integrated into an existing polymer-metal biosensor platform. While the equivalent circuit was more complex than an unconjugated IDE alone, it was found that similar emphasis could be placed on the 1 MHz-4 MHz region for this application, based on fitting and Nyquist analysis. Moreover, a linear regression was performed, resulting in a high correlation for this application as well (*R*^2^ value of 0.90517) based on the high-frequency Faradaic (kinetic) region. While the results did demonstrate reliability, the robust and repeatable binding of the antibody can be further improved by conjugation chemistry should a specific orientation of the antibody be desired in future research. Overall, the trend observed was an increase in impedance with increasing L-glutamine concentrations.

In both IDE experiments described, equivalent circuit and Nyquist analysis enabled the acquisition of multifarious datasets from impedimetric sensors integrated into an existing biosensor platform in addition to electrophysiology and transparency-mediated assays.

Due to its optical properties, the transparent polymer provided an ample basis for calcein AM/propidium iodide staining without interference and additionally demonstrated the biocompatibility of the platform. The microfluidics integration was demonstrated by the process optimization of micromilling of the substrate and replacement of the 3D microelectrode structures with microfluidic ports. Through COMSOL finite element modeling, localized fluid injection was additionally demonstrated with these 3D ports, which may be applied in localized chemical stimulation, nutrient delivery, or even organoid fixation in culture^5^.

Together, this work serves to demonstrate accessible approaches for the development of polymer-metal fabrications for biosensor applications and how impedimetric data coupled with equivalent circuit analysis can result in novel multifarious data from platform technology. The authors acknowledge that all modalities described herein are not utilized in a combinatorial fashion; however, interested readers may find them practically utilized and described in other collaborative works from our group^5,13,35^.

## Supplementary information

Supplemental Materials

## References

[1] Anderson WA, Bosak A, Hogberg HT, Hartung T, Moore MJ (2021). Advances in 3D neuronal microphysiological systems: towards a functional nervous system on a chip. In Vitro Cell. Dev. Biol. -Anim..

[2] Kundu A (2021). Fabrication and characterization of 3D printed, 3D microelectrode arrays for interfacing with a peripheral nerve-on-a-chip. ACS Biomater. Sci. Eng..

[3] Bhatia SN, Ingber DE (2014). Microfluidic organs-on-chips. Nat. Biotechnol..

[4] Didier CM, Kundu A, Deroo D, Rajarman S (2020). Development of in vitro 2D and 3D microelectrode arrays and their role in advancing biomedical research. J. Micromech. Microeng..

[5] Orrico, J. F. et al. In *2021 21st International Conference on Solid-State Sensors, Actuators and Microsystems (Transducers)*. 767–770 (IEEE, 2021).

[6] Park Y (2021). Three-dimensional, multifunctional neural interfaces for cortical spheroids and engineered assembloids. Sci. Adv..

[7] Zhang B, Korolj A, Lai BFL, Radisic M (2018). Advances in organ-on-a-chip engineering. Nat. Rev. Mater..

[8] Cong Y (2020). Drug toxicity evaluation based on organ-on-a-chip technology: a review. Micromachines.

[9] Didier, C. M., Kundu, A. & Rajaraman, S. Facile, Packaging substrate-agnostic, microfabrication and assembly of scalable 3D metal microelectrode arrays for in vitro organ-on-a-chip and cellular disease modeling. *20th International Conference on Solid-State Sensors, Actuators and Microsystems & Eurosensors XXXIII (TRANSDUCERS & EUROSENSORS XXXIII)*, 1686-1689, 10.1109/TRANSDUCERS.2019.8808364 (2019).

[10] Susloparova A (2021). Low impedance and highly transparent microelectrode arrays (MEA) for in vitro neuron electrical activity probing. Sens. Actuat. B: Chem..

[11] Schmidt S, Frank R, Krinke D, Jahnke H-G, Robitzki AA (2022). Novel PMMA based 96-well microelectrode arrays for bioelectronic high throughput monitoring of cells in a live mode. Biosens. Bioelectron..

[12] Gondosiswanto R, Hibbert DB, Fang Y, Zhao C (2018). Redox recycling amplification using an interdigitated microelectrode array for ionic liquid-based oxygen sensors. Anal. Chem..

[13] Didier CM, Kundu A, Shoemaker JT, Vukasinovic J, Rajaraman S (2020). SeedEZ™ interdigitated electrodes and multifunctional layered biosensor composites (MLBCs): a paradigm shift in the development of in vitro biomicrosystems. J. Microelectromech Syst..

[14] Didier, C. M., Kundu, A., Castro, J. M., Hart, C. & Rajaraman, S. Compact Micro-Stereolithographic (µSLA) Printed, 3D Microelectrode Arrays (3D MEAS) with Monolithically Defined Positive and Negative Relief Features For in Vitro Cardiac Beat Sensing. *2022 IEEE 35th International Conference on Micro Electro Mechanical Systems Conference (MEMS)*, 325-328, 10.1109/MEMS51670.2022.9699662 (2022).

[15] Choi JS, Lee HJ, Rajaraman S, Kim D-H (2021). Recent advances in three-dimensional microelectrode array technologies for in vitro and in vivo cardiac and neuronal interfaces. Biosens. Bioelectron..

[16] Hart C, Kumar KS, Li J, Thomas J, Rajaraman S (2020). Investigation of the Enhanced Sensitivity of Interdigitated Electrodes for Cellular Biosensing With Geometric, Nanostructured Surface Area, and Surface Plasmon Resonance Modes. J. Microelectromech. Syst..

[17] Reverter, F. et al. Design considerations for a CMOS Lab-on-Chip microheater array to facilitate the in vitro thermal stimulation of neurons. *2014 IEEE International Symposium on Circuits and Systems (ISCAS)*, p. 630–633, 10.1109/ISCAS.2014.6865214 (2014).

[18] Kondzior M, Grabowska I (2020). Antibody-electroactive probe conjugates based electrochemical immunosensors. Sensors.

[19] Nieto D, McGlynn P, de la Fuente M, Lopez-Lopez R, O’Connor GM (2017). Laser microfabrication of a microheater chip for cell culture outside a cell incubator. Colloids Surf. B Biointerfaces.

[20] Rusli, N. I., Espinar, P. L., Ceyssens, F., Taurino, I. & Kraft, M. Miniaturized electrochemical device for in-situ monitoring of glucose, lactate, dissolved oxygen, PH, and temperature in yeast culture. *2021 21st International Conference on Solid-State Sensors, Actuators and Microsystems (Transducers)*, 188-191, 10.1109/Transducers50396.2021.9495611 (2021).

[21] Baldwin A, Yu L, Meng E (2016). An electrochemical impedance-based thermal flow sensor for physiological fluids. J. Microelectromech. Syst..

[22] Didier, C. M. et al. Polymer and stainless steel-based 3d microelectrode arrays (3D meas), with penta-modal sensing capabilities for the investigation of electrognic cells. *Proceedings of the 2022 Hilton Head MEMS Workshop* (2022).

[23] Franks W, Schenker I, Schmutz P, Hierlemann A (2005). Impedance characterization and modeling of electrodes for biomedical applications. IEEE Trans. Biomed. Eng..

[24] Vani K (2013). In vitro biocompatiblity of modified polycarbonate as a biomaterial. Colloids Surf. B: Biointerfaces.

[25] Mo L (2017). On the temperature dependency and reversibility of sheet resistance of silver nanoparticles covered by 3-mercaptopropionic acid. J. Mater. Sci.: Mater. Electron..

[26] Rajaraman, S. *Micromachined Three-dimensional Electrode Arrays For In-vitro And In-vivo Electrogenic Cellular Networks* (Georgia Institute of Technology, 2009).

[27] Didier, C. *Development of 3D Printed and 3D Metal-Based Micro/Nanofabricated, and Nano-Functionalized, Microelectrode Array (MEA) Biosensors For Flexible, Conformable, and In Vitro Applications* (University of Central Florida, 2019).

[28] Wang Z, Murphy A, O’Riordan A, O’Connell I (2021). Equivalent impedance models for electrochemical nanosensor-based integrated system design. Sensors.

[29] Jorcin J-B, Orazem ME, Pébère N, Tribollet B (2006). CPE analysis by local electrochemical impedance spectroscopy. Electrochim. Acta.

[30] Castro, J. M. & Rajaraman, S. Constant phase element (CPE) modeling and analysis of multi-material, micro-bullet shaped, high-throughput 3D microelectrodes for in-vitro electrophysiological applications. *Proceedings of the 2022 Hilton Head MEMS Workshop* (2022).

[31] Bondarenko, A. S. & Ragoisha, G. A. *EIS Spectrum Analyser - Equivalent Circuit Elements and Parameters*, http://www.abc.chemistry.bsu.by/vi/analyser/parameters.html (2008).

[32] Ragoisha GA, Bondarenko AS (2005). Potentiodynamic electrochemical impedance spectroscopy. Electrochim. Acta.

[33] Lasia, A. *Electrochemical Impedance Spectroscopy And Its Applications*. *Modern aspects of electrochemistry*, 67–125 (Springer Nature, 2002).

[34] Leung TKW (2021). Micro‐electrodes for in situ temperature and bio‐impedance measurement. Nano Sel..

[35] Didier, C. M. et al. Fully integrated 3D microelectrode arrays with polydopamine-mediated silicon dioxide insulation, for electrophysiological interrogation of a novel 3D human, neural microphysiological construct. *In**ACS Applied Materials & Interfaces* (American Chemical Society, 2022). 10.1021/acsami.3c05788 37494582

[36] Uygun ZO, Ertuğrul Uygun HD (2014). A short footnote: circuit design for faradaic impedimetric sensors and biosensors. Sens. Actuat. B: Chem..

[37] Borkholder, D. *Cell Based Biosensors Using Microelectrodes* (Stanford University, 1998).

[38] Inkpen MS (2019). Non-chemisorbed gold–sulfur binding prevails in self-assembled monolayers. Nat. Chem..

[39] Bürgi T (2015). Properties of the gold–sulphur interface: from self-assembled monolayers to clusters. Nanoscale.

[40] Karyakin AA, Presnova GV, Rubtsova MY, Egorov AM (2000). Oriented immobilization of antibodies onto the gold surfaces via their native thiol groups. Anal. Chem..

[41] Ruiz G, Ryan N, Rutschke K, Awotunde O, Driskell JD (2019). Antibodies irreversibly adsorb to gold nanoparticles and resist displacement by common blood proteins. Langmuir.

[42] Eagle H, Oyama VI, Levy M, Horton CL, Fleischman R (1956). The growth response of mammalian cells in tissue culture to L-glutamine and L-glutamic acid. J. Biol. Chem..

[43] Jagušić M (2016). Stability of Minimum Essential Medium functionality despite L-glutamine decomposition. Cytotechnology.

[44] Rubin H (2019). Deprivation of glutamine in cell culture reveals its potential for treating cancer. Proc. Natl Acad. Sci. USA.

[45] MacKay S (2017). Using impedance measurements to characterize surface modified with gold nanoparticles. Sensors.

[46] Strong ME, Richards JR, Torres M, Beck CM, La Belle JT (2021). Faradaic electrochemical impedance spectroscopy for enhanced analyte detection in diagnostics. Biosens. Bioelectron..

[47] Kaigala GV, Lovchik RD, Delamarche E (2012). Microfluidics in the “open space” for performing localized chemistry on biological interfaces. Angew. Chem. Int. Ed..

[48] Zibek S, Hagmeyer B, Stett A, Stelzle M (2010). Chemical stimulation of adherent cells by localized application of acetylcholine from a microfluidic system. Front. Neuroeng..

[49] Hart C, Rajaraman S (2020). Low-power, multimodal laser micromachining of materials for applications in sub-5 µm shadow masks and sub-10 μm interdigitated electrodes (IDEs) fabrication. Micromachines.

[50] Castro, J. M. & Rajaraman, S. Experimental and modeling based investigations of process parameters on a novel, 3d printed and self-insulated 24-well, high-throughput 3d microelectrode array device for biological applications. *J. Microelectromech. Syst.* **31.3**, 358–371 (2022).

