# Supplementary material for: Microfabricated polymer-metal biosensors for multifarious data collection from electrogenic cellular models
Source: Microsyst Nanoeng. 2023 Mar 1;9:22. doi: 10.1038/s41378-023-00488-1 (PMC9974480; doi:10.1038/s41378-023-00488-1)
Supplement: Supplementary file 1 — Supplemental Materials [file 41378_2023_488_MOESM1_ESM.docx]

**MICROFABRICATED POLYMER-METAL BIOSENSORS FOR MULTIFARIOUS DATA COLLECTION FROM ELECTROGENIC CELLULAR MODELS**

Charles M Didiera,b,Ϯ, Julia F. Orricoa,Ϯ, Omar S Cepeda-Torresa,c,Ϯ, Jorge Manrique Castroa,d, Aliyah Baksha, Swaminathan Rajaramana,b,d,e,*

*aNanoScience Technology Center, University of Central Florida, 4353 Scorpius Street, Research I, Suite 231, FL, 32816, Orlando, USA*

*bBurnett School of Biomedical Sciences, University of Central Florida, 6900 Lake Nona Blvd, FL, 32827,*

*Orlando, USA*

*cDepartment of Biomedical Engineering, Polytechnic University of Puerto Rico, 377, 00918, Ponce de Leon, San Juan, Puerto Rico*

*dDepartment of Electrical and Computer Engineering, University of Central Florida, 4238 Scorpius Street, FL, 32816, Orlando, USA*

*eDepartment of Materials Science and Engineering, University of Central Florida, 12760 Pegasus Drive, Engineering I, Suite 207, FL, 32816, Orlando, USA*

# Supplementary Information

# Additional data pertaining to material biocompatibility and transparency, along with temperature differential recordings, analyte differential recording and expanded microfluidic characterization from the main text may be found below.

# *Material Biocompatibility and Cellular Imaging*

# Figure S1 presents the brightfield and fluorescent confocal microscopy images of the C2C12 myocytes grown on the polymer-metal platform approach detailed in this work. To assess the transparency of the substrate after functionalization (without attachment of the microheater), optical microscopy was utilized, additionally with Calcein AM and Propidium Iodide counterstaining. Some background fluorescence is observed, however cell populations are still readily observed. From the fluorescence data obtained, a cell viability of >97% was calculated, which demonstrates high biocompatibility of the culture, indicative of a suitable material set (including such non-traditional integrations such as Ag-ink) and fabrication processes.

# *Differential Temperature Data*

# Figure S2 here presents the overall fitted Nyquist data for the temperature sensing IDE found in the main text. The equivalent circuit utilized for fitting is again included for reference, and the included Table S1 contains the relevant parameters derived from the plotted fittings for each of the selected temperature states. Additionally, the phase plot for all tested temperatures is included for reference.

# *Differential Conjugation Data*

# Figure S3 presents the overall fitted Nyquist data for the analyte sensing IDE found in the main text. The equivalent circuit utilized for fitting the analyte IDE sensor is included for reference. Again, the circuit utilized in the temperature sensing IDE was similarly utilized to fit the IDE here as well, before any antibody conjugation. Table S2 contains the relevant parameters derived from the plotted fittings for each of the states of the IDE. Finally, the phase plot for the tested conditions has been included for reference.

# *COMSOL Model Data*

# Figure S4 here presents additional time points for the COMSOL fluidic modelling from the main body of work. The additional time points include: 5s, 10s, 15s, and 20s. These additional times are performed under the same conditions outlined in the main text and are performed with a continued application of 8Pa of pressure. As is shown in Figure S3, as time continues, the 0.2mM L-Glutamine perfuses further into the culture area, but remains localized centrally due to the continued application of force in the inlet port. Such longer time points under continued pressure serve to illustrate how these microfluidic ports could be utilized for nutrient re-addition in a timely manner to for instance supplement cell culture media, instead of performing a full media change if desired.

#
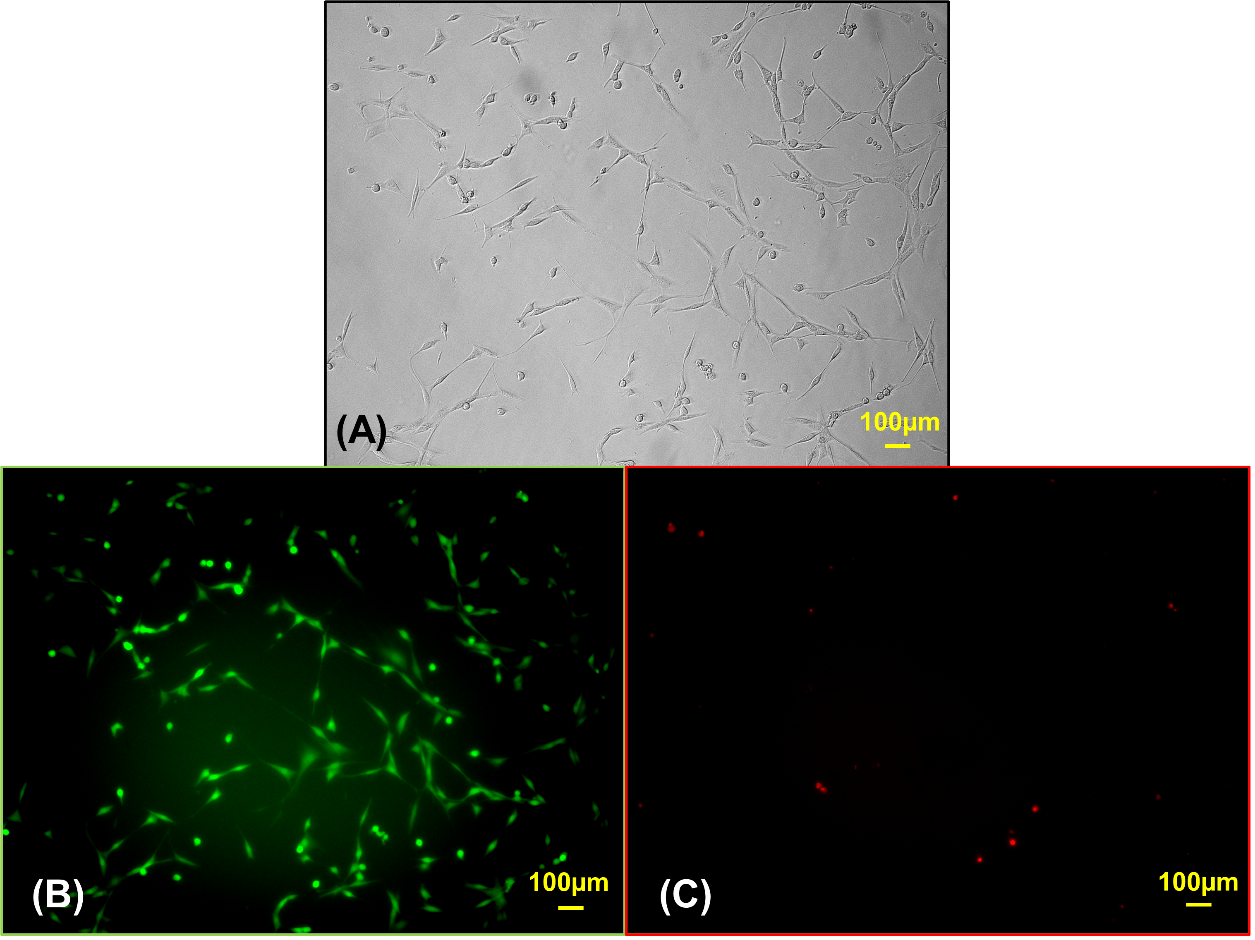


***Figure S1:*** *(A-C) Optical and fluorescent imaging of C2C12 myocyte cells on the device at 5 DIV, demonstrating the optical clarity of the fabricated device, as well as excellent cell viability. (A) Optical imaging of the C2C12 myocytes. (B) Calcein AM, live staining of the cells. (C) Propidium Iodide stain, for live/dead cell confirmation.*


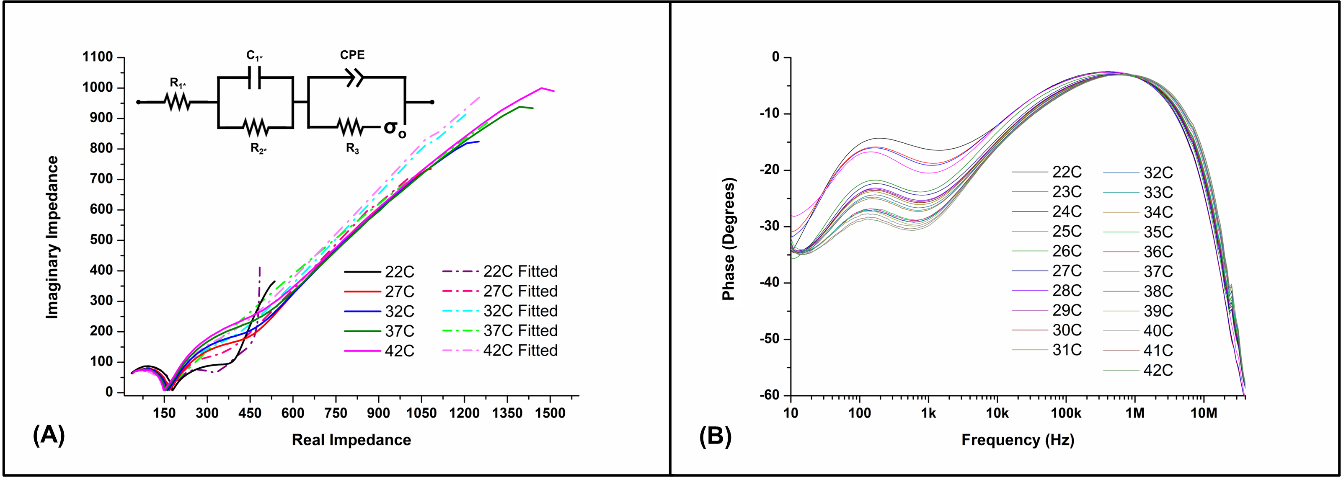


***Figure S2:*** *Additional graphical information for the temperature sensing IDE. (A) Full Nyquist plot for the fitted and experimental results for the selected temperatures. The inset includes the equivalent circuit for reference. (B) Full spectrum phase plot for all temperatures.*


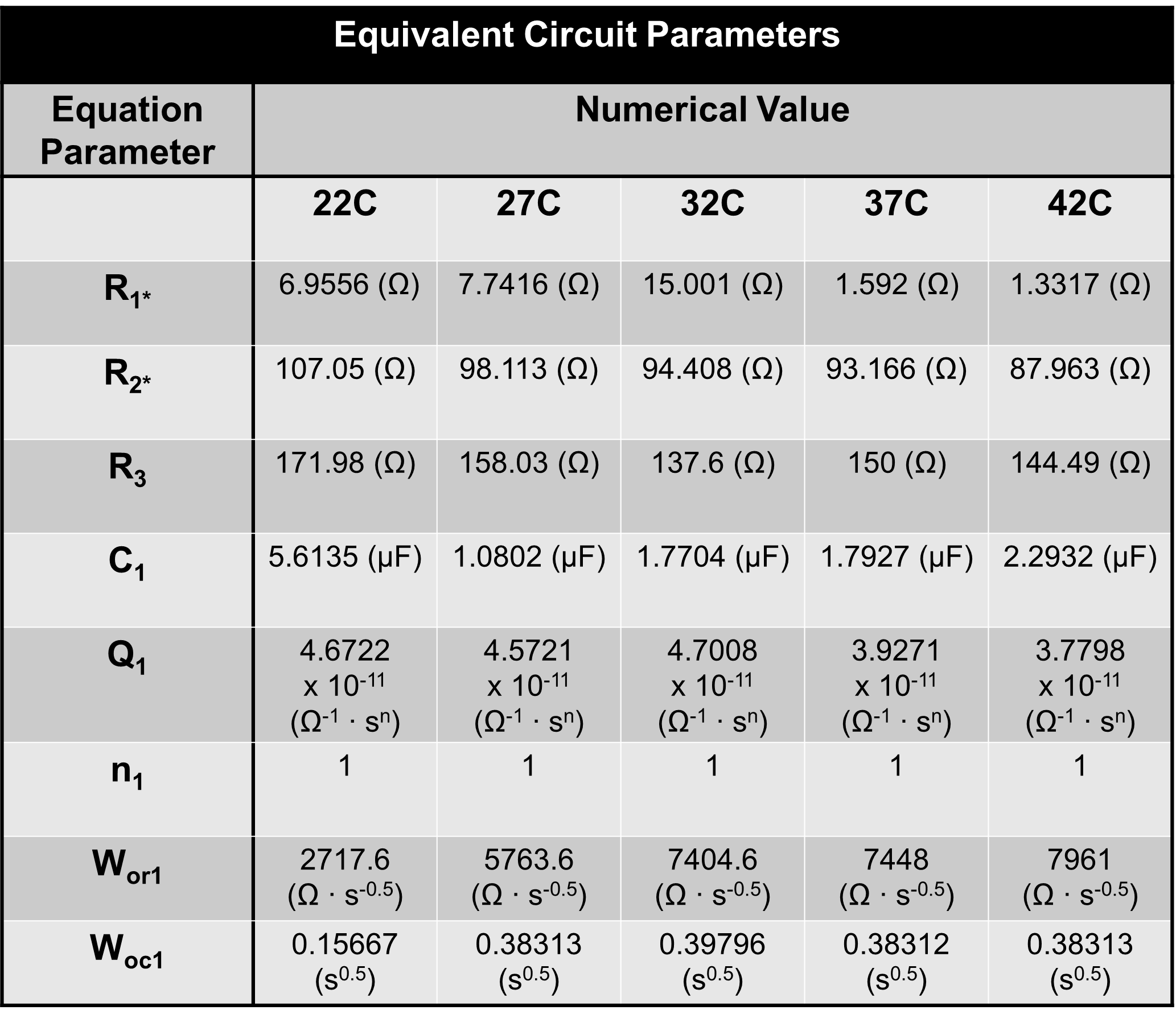


***Table S1:*** *Extracted equivalent circuit parameters from temperature modelled IDE.*


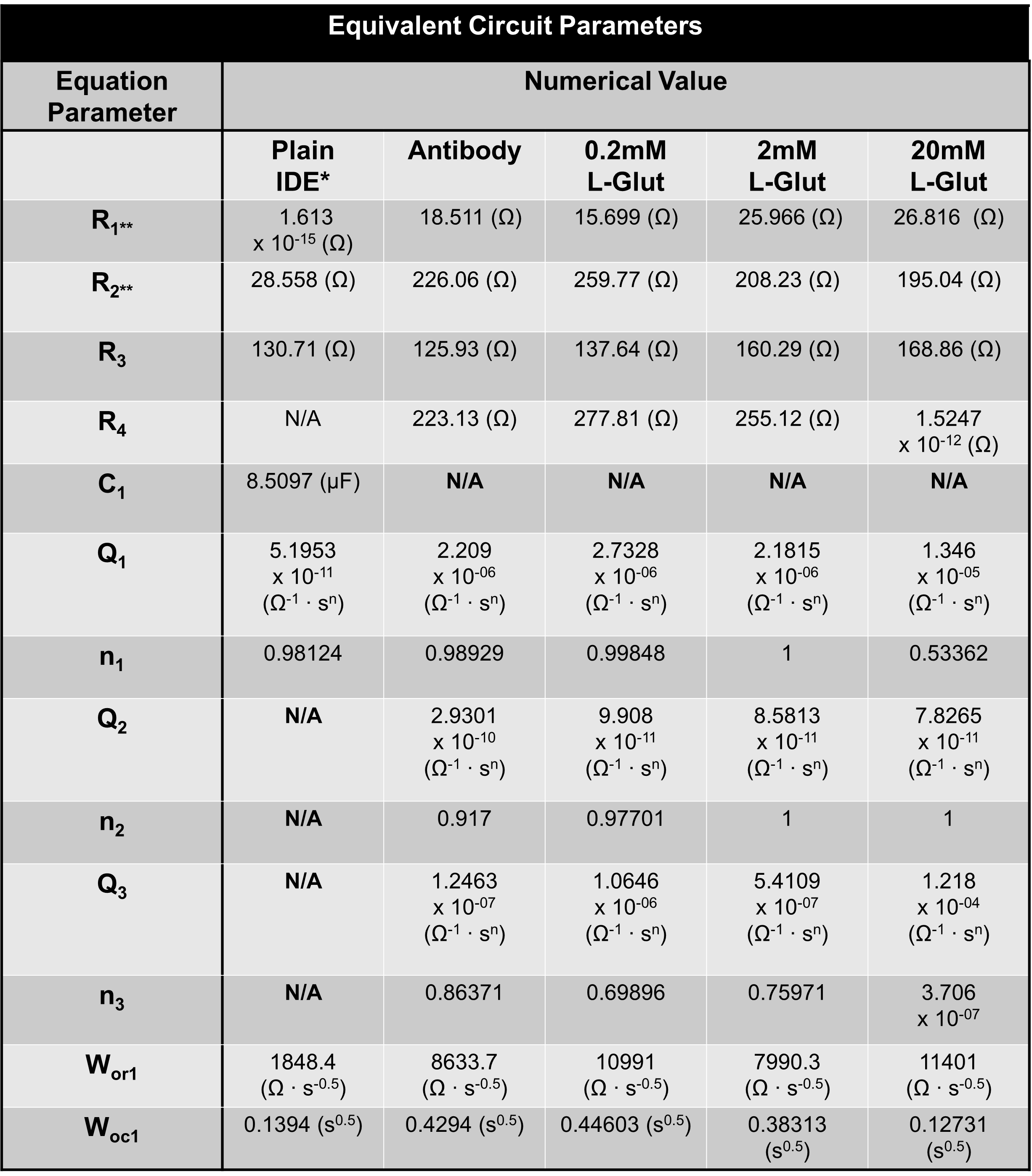


***Table S2:*** *Extracted equivalent circuit parameters from analyte modelled IDE.*

***Figure S3:*** *Additional graphical information for the analyte sensing IDE. (A) Full Nyquist plot for the fitted and experimental results for the tested conditions. The inset (for reference) includes the equivalent circuit of the conjugated sensor, and for the tested concentrations of L-Glutamine. (B) Full spectrum phase plot for all tested conditions.*


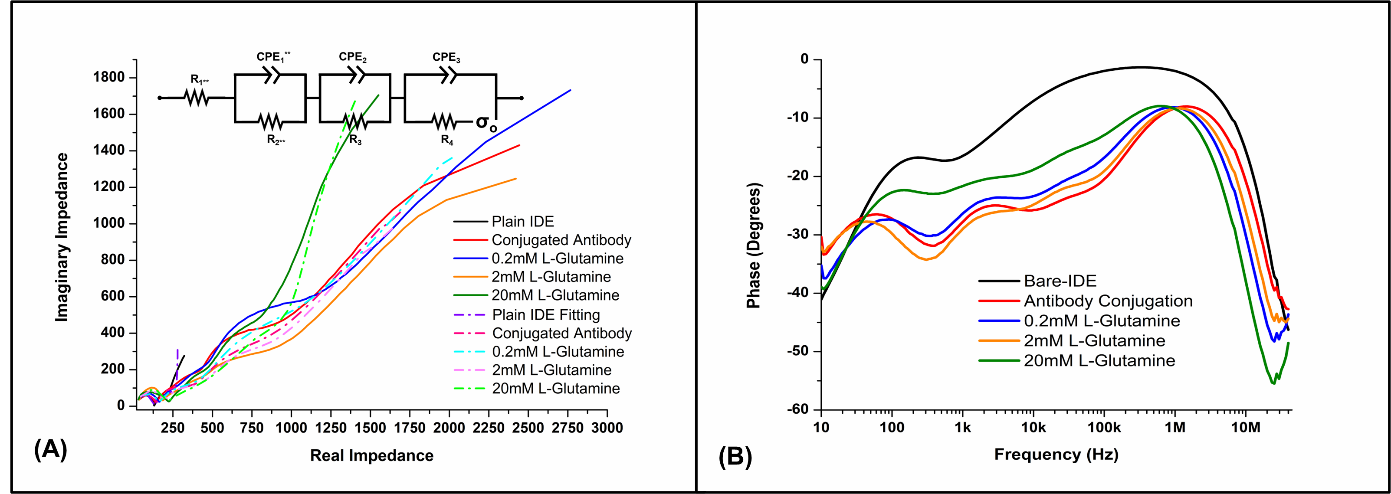

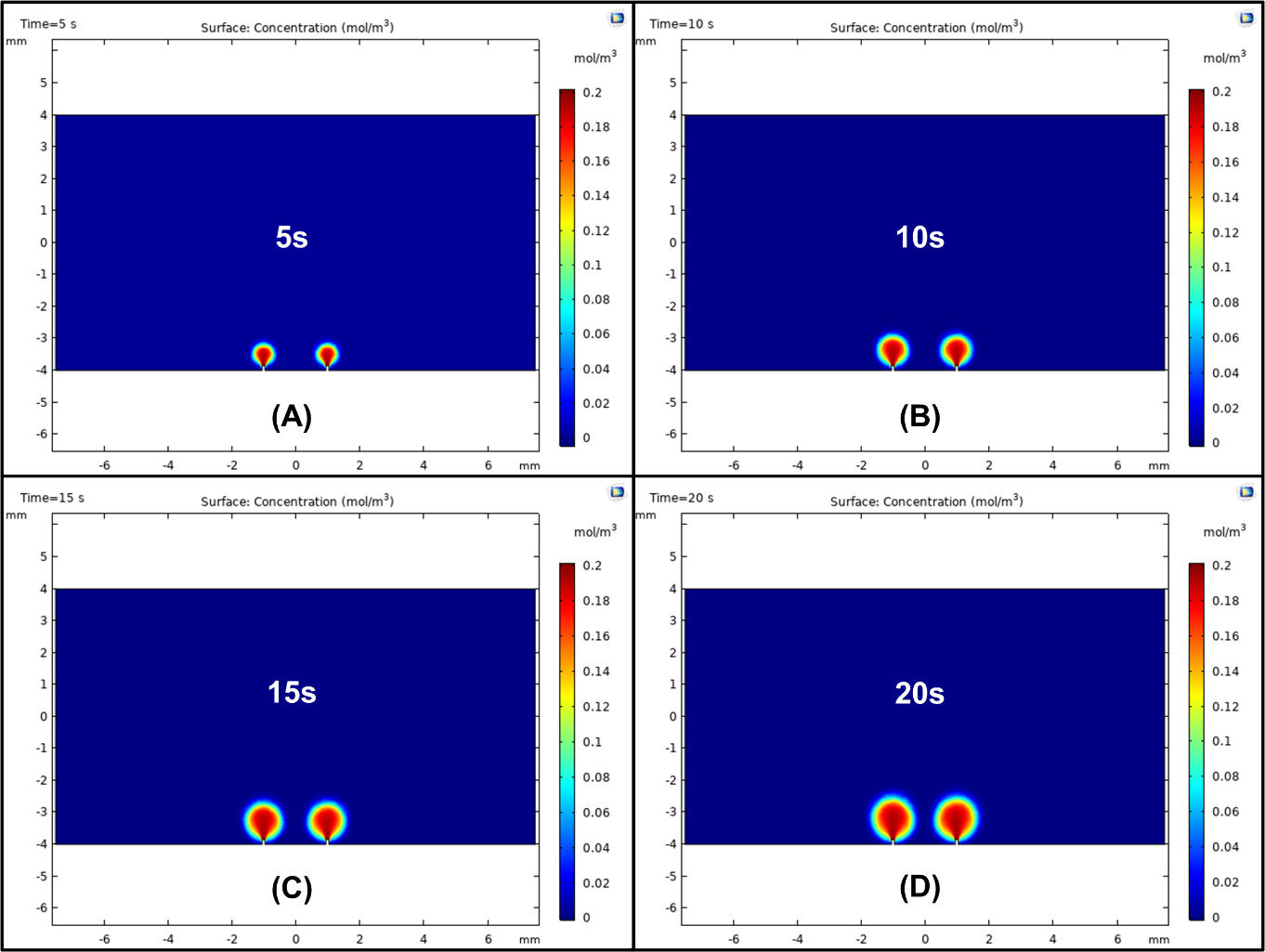


***Figure S4:*** *Additional time points for the COMSOL fluidic model, with continuously applied 8Pa pressure on the inlets. (A-E) Time points are: 5s (A), 10s (B), 15s (C), and 20s (D) respectively.*
